# Supplementary material for: Red Blood Cell-Derived Microparticles Exert No Cancer Promoting Effects on Colorectal Cancer Cells In Vitro
Source: Int J Mol Sci. 2022 Aug 18;23(16):9323. doi: 10.3390/ijms23169323 (PMC9409112; doi:10.3390/ijms23169323)
Supplement: Supplementary file 1 [file ijms-23-09323-s001.zip › ijms-1863930-supplementary/ijms-1863930-supplementary figures.pdf]

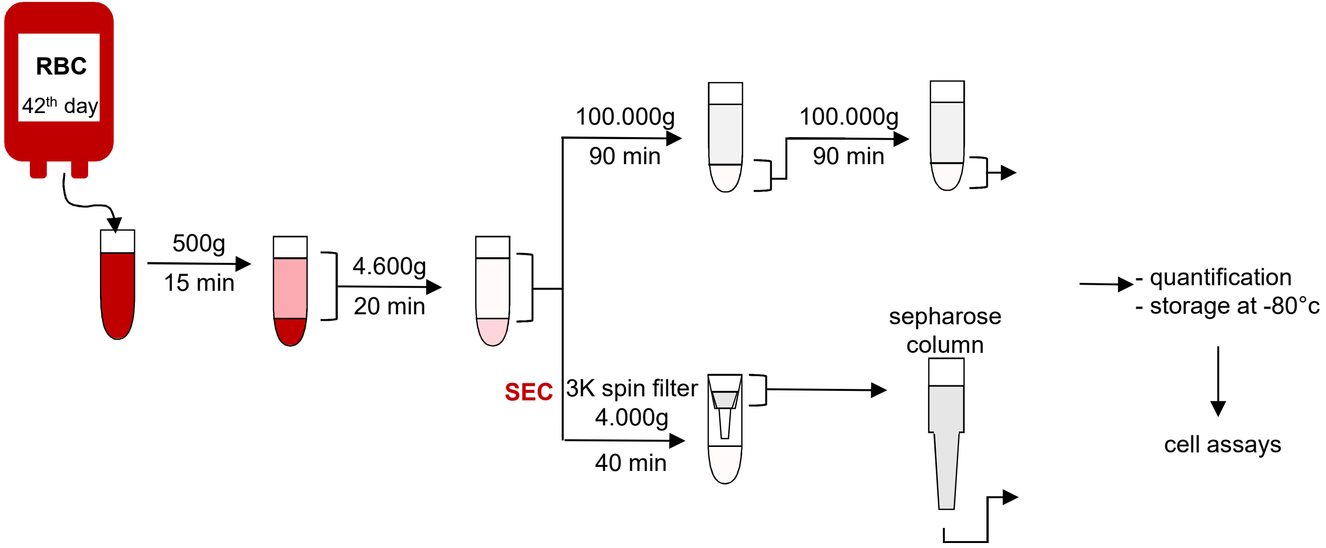

Figure S1: Scheme for RMP isolation and processing

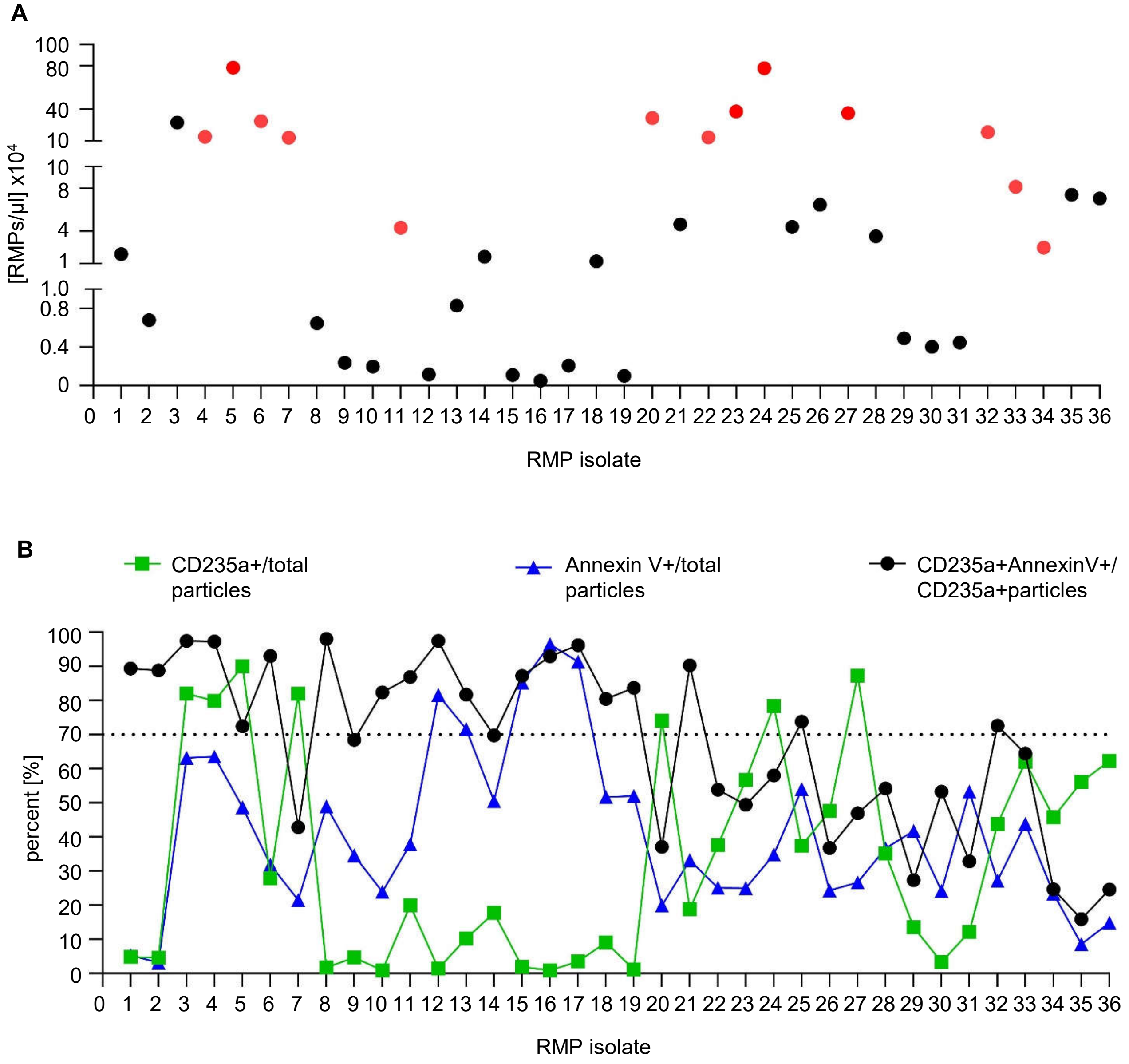

Figure S2: RMP concentration and relative distribution of CD235a and/or Annexin V positive particles for each RMP isolate. Presented are the RMP concentrations (A) and the relative distributions of CD235a and/or Annexin V positive particles (B) of 31 UC (no. 1-31) and 5 SEC (no. 32-36) isolates. Red dots (A) indicate RMP isolate used in either viability, proliferation, migration/invasion, reportergene assay or western blot analysis. *RMP, red blood-derived microparticle; SEC, size exclusion chromatography; UC, ultracentrifugation.*

isolate	1	2	3	4	5	6	7	8	9	10
RMPs/CD235a+ [%]	89	89	97	97	72	93	43	98	68	82
[RMPs/µl]	18.496	6.769	273.000	140.000	784.000	287.000	134.000	6.453	2.360	1.980

isolate	11	12	13	14	15	16	17	18	19	20
RMPs/CD235a+ [%]	87	97	82	70	87	93	96	80	84	37
[RMPs/µl]	43.166	1.168	8.269	16.325	1.089	528	2.072	11.972	1.004	314.777

isolate	21	22	23	24	25	26	27	28	29	30
RMPs/CD235a+ [%]	90	54	49	58	74	37	47	54	27	53
[RMPs/µl]	46.414	136.565	375.584	779.997	44.146	64.557	360.992	35.230	4.909	4.010

isolate	31	32	33	34	35	36
RMPs/CD235a+ [%]	33	73	64	25	16	25
[RMPs/µl]	4.464	182.566	81.405	24.765	74.050	70.431

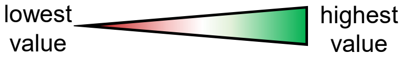

Figure S3: Comparison of the CD235a and Annexin V (RMP)/CD235a ratio to the RMP concentration per isolate. Presented are the ratios of 31 UC (no. 1-31) and 5 SEC isolates (no. 32-36) in percent. The green and red color scaling highlights the highest and the lowest values of the RMP/CD235a ratio and RMP concentration, respectively. *RMP, red blood-derived microparticle; SEC, size exclusion chromatography; UC, ultracentrifugation.*

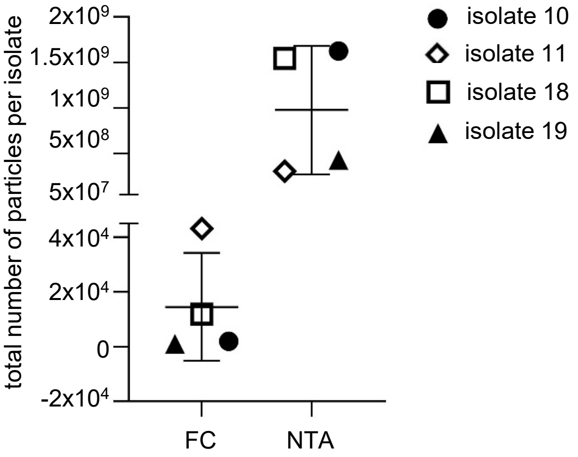

Figure S4: Influence of the quantification method on particle number per isolate. The particle concentration of four UC isolates were determined via flow cytometry and nano particle tracking analysis. Each symbol represents a different isolation. Data is presented as mean ± SD (n=4). *FC, flow cytometry; NTA, nanoparticle tracking analysis.*

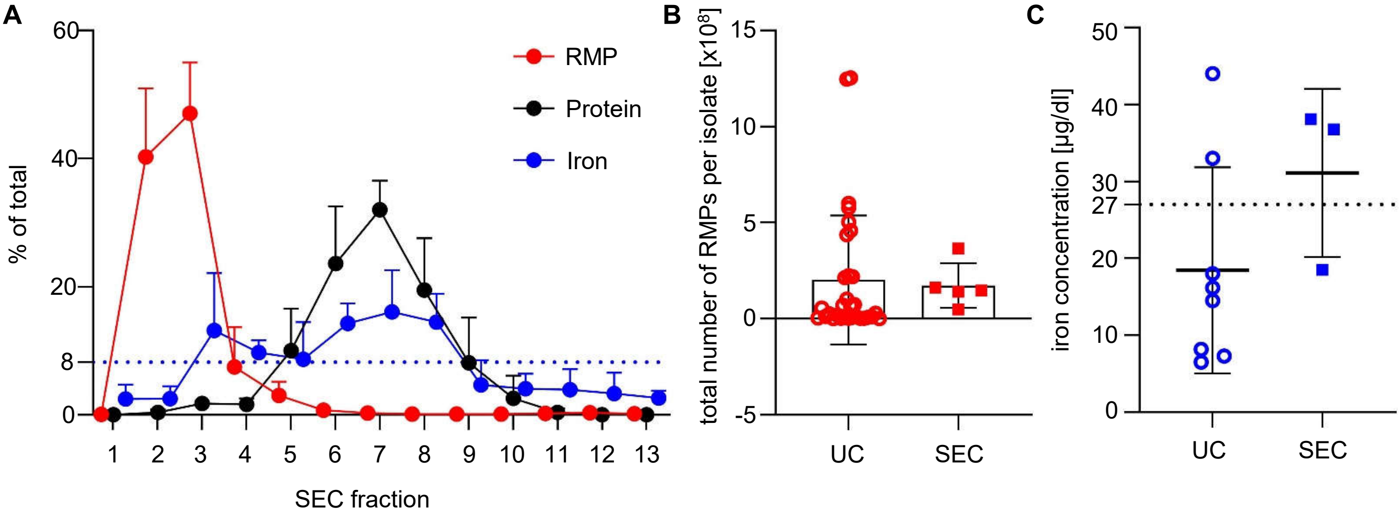

Figure S5: Characterization of RMPs isolated via SEC. (A)The mean values + SD of three independent SEC isolations (n=3) are presented as % of total number that passed the column. Red dots: RMP concentration of each fraction measured via flow cytometry (CD2351 and Annexin V positive vesicles). Black dots: Protein concentration of each fraction measured via bicinchoninic acid (BCA) protein assay. Blue dots: Iron content of each fraction measured by QuantiChrom™ Iron Assay. Notably, iron concentration values of fraction 1, 2, 9-13 are under the assay detection limit of 27 µg/dl as highlighted by the dashed blue line. (B, C) Comparison of the RMP and iron concentration of UC and SEC isolates. (B) RMP amount per isolate from 31 UC and 5 SEC isolations. (C) Iron concentration of RMP isolates from 8 UC and 3 SEC isolations. The dashed line represents the assay detection limit of 27 µg/dl. Data is presented as mean ±SD. *RMP, red blood-derived microparticle; SEC, size exclusion chromatography; UC, ultracentrifugation.*

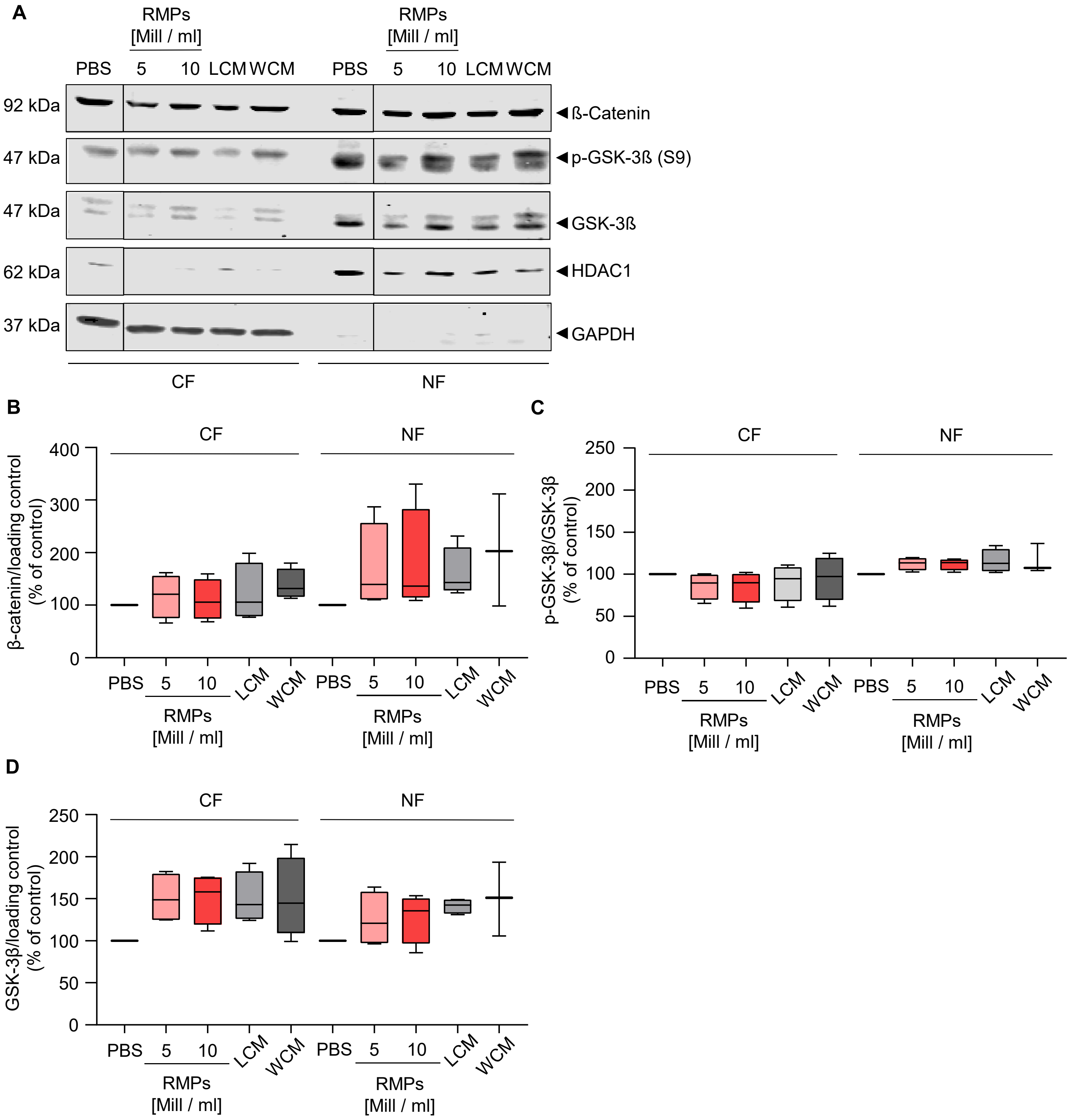

Figure S6: Effect of red-blood cell derived microparticles on Wnt signaling, in cellular and nuclear fractions of HCT-116 cells. HCT-116 cells were incubated with two concentrations of RMPs (5 x 10^6 or 10 x 10^6 particles / ml), vehicle control (PBS), L-cell conditioned medium (LCM; negative control) and Wnt-3A conditioned medium (WCM; containing active Wnt-3A protein) as positive control . After 24 hours, cells were lysed and cytosolic and nuclear fractions (CF, NF) were prepared. Protein level of β-catenin, p-GSK-3β and GSK-3β, were assessed by western blot analysis. GAPDH and HDAC-1 were used as loading control for cytosolic and nuclear fractions, respectiveley. (A) One representative western blot is shown out of four independent experiments (n=4) (B-E) Quantification of β-catenin (B), p-GSK-3β (C) and GSK-3β (D) level after normalization to either loading control (B, D) or GSK-3β (C). Data represents median, 25th to 75th percentile (box), minimum to maximum (whiskers) versus vehicle control (n=4).

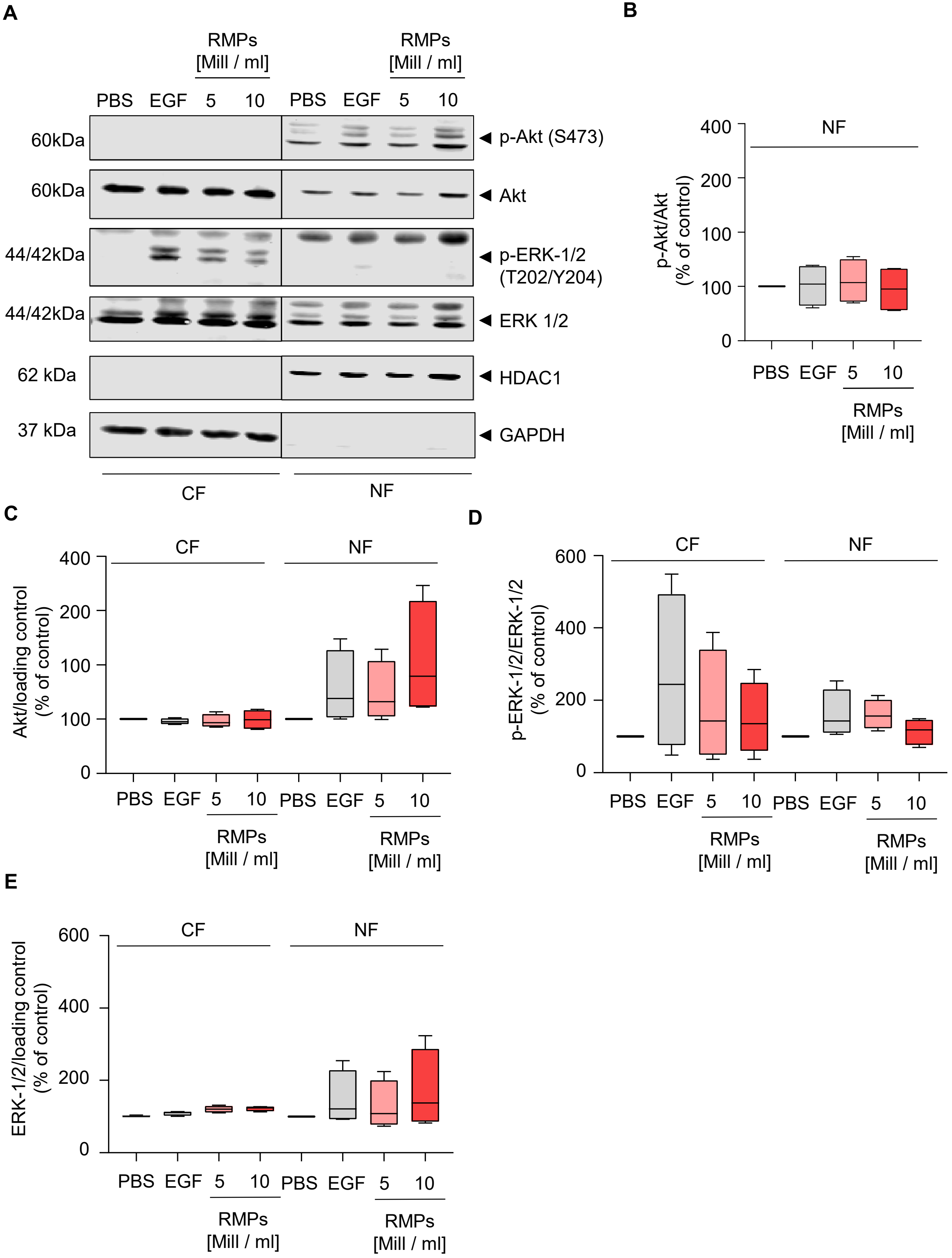

Figure S7: Effect of red-blood cell derived microparticles on Akt and ERK signaling in cellular and nuclear fractions of HCT-116 cells. HCT-116 cells were incubated with two concentrations of RMPs (5 x 10^6 or 10 x 10^6 particles / ml), vehicle control (PBS) and EGF (50 ng/ml) as positive control . After 24 hours (RMP and PBS treatment) or 30 min (EGF treatment), cells were lysed and cytosolic and nuclear fractions were prepared. Protein level of p-Akt, Akt, pERK-1/2 and ERK were assessed by western blot analysis. GAPDH and HDAC-1 were used as loading control for cytosolic and nuclear fractions, respectiveley. (A) One representative western blot is shown out of four independent experiments (n=4) (B-E) Quantification of p-Akt (B), Akt (C), p-ERK-1/2 (D) and ERK-1/2 (E) level after normalization to either loading control (C, E), Akt (B) or ERK-1/2 (D). Data represents median, 25th to 75th percentile (box), minimum to maximum (whiskers) versus vehicle control (n=4).
