# Supplementary material for: Red Blood Cell-Derived Microparticles Exert No Cancer Promoting Effects on Colorectal Cancer Cells In Vitro
Source: Int J Mol Sci. 2022 Aug 18;23(16):9323. doi: 10.3390/ijms23169323 (PMC9409112; doi:10.3390/ijms23169323)
Supplement: Supplementary file 1 [file ijms-23-09323-s001.zip › ijms-1863930-supplementary/ijms-1863930-supplementary Table S1.pdf]

## Supplementary methods:

**Supplementary Table S1**: detailed antibody details

| antibody/reagent | application | amount /test | antibody dilution reagent | source | cat. no | company |
|---|---|---|---|---|---|---|
| Anti-CD235a-BV421 | FC | 2 µl | PBS | mouse | 562938 | BD Bioscience, Franklin Lakes, NJ, USA |
| Annexin V APC | FC | 10 µl | PBS | n.a. | 550474 | BD Bioscience, Franklin Lakes, NJ, USA |
| Anti-GSK-3β (L-17) | WB | 1:1,000 | 1% BSA in TBS | goat | sc-8257 | Santa Cruz Biotechnology, Dallas, TX, USA |
| Anti-p-GSK-3β (Ser9) | WB | 1:500 | 1% BSA in TBS | goat | sc-11757 | Santa Cruz Biotechnology, Dallas, TX, USA |
| Anti-PCNA | WB | 1:1,000 | Intercept buffer 1:1 in TBST | mouse | ab29 | Abcam, Cambridge, UK |
| Anti-β-actin | WB | 1:1,000 | 1% BSA in TBST | mouse | sc-47778 | Santa Cruz Biotechnology, Dallas, TX, USA |
| Anti-p-Akt (Ser473) | WB | 1:1,000 | 1% BSA in TBST | rabbit | 9271S | Cell Signaling Technology, Dancer, MA, USA |
| Anti-Akt | WB | 1:1,000 | 1% BSA in TBST* | rabbit | 9272S | Cell Signaling Technology, Dancer, MA, USA |
| Anti-p-ERK-1/2 (pT202/pY204.22A) | WB | 1:1,000 | 1% BSA in TBST* | mouse | sc-136521 | Santa Cruz Biotechnology, Dallas, TX, USA |
| Anti-ERK-1/2 (C-9) | WB | 1:1,000 | 1% BSA in TBST* | mouse | sc-514302 | Santa Cruz Biotechnology, Dallas, TX, USA |
| Anti-β-Catenin (E-5) | WB | 1:1,000 | 1% BSA in TBST* | mouse | sc-7963 | Santa Cruz Biotechnology, Dallas, TX, USA |
| Anti-GAPDH | WB | 1:1,000 | 1% BSA in TBST* | rabbit | ab9485 | Abcam, Cambridge, UK |
| Anti-HDAC-1 (10E2) | WB | 1:1,000 | 1% BSA in TBST* | mouse | 5356S | Cell Signaling Technology, Dancer, MA, USA |
| 800CW donkey anti-goat | WB | 1:10,000 1:20,000 | 1% BSA in TBST* | donkey | 926-32214 | LI-COR Biosciences, Lincoln, NE, USA |
| 680RD donkey anti-goat | WB | 1:10,000 1:20,000 | 1% BSA in TBST* | donkey | 926-68074 | LI-COR Biosciences, Lincoln, NE, USA |
| 800CW donkey anti-mouse | WB | 1:10,000 1:20,000 | 1% BSA in TBST* | donkey | 926-32212 | LI-COR Biosciences, Lincoln, NE, USA |
| 800CW donkey anti-rabbit | WB | 1:10,000 1:20,000 | 1% BSA in TBST* | donkey | 926-32213 | LI-COR Biosciences, Lincoln, NE, USA |
| 680RD donkey anti-mouse | WB | 1:10,000 1:20,000 | 1% BSA in TBST* | donkey | 926-68072 | LI-COR Biosciences, Lincoln, NE, USA |
| 680RD donkey anti-rabbit | WB | 1:10,000 1:20,000 | 1% BSA in TBST* | donkey | 926-68073 | LI-COR Biosciences, Lincoln, NE, USA |

*TBST: TBS with 0.01% Tween-20
